# Proteomic and Phosphoproteomic Analysis Reveals that Neurokinin-1 Receptor (NK1R) Blockade with Aprepitant in Human Keratinocytes Activates a Distinct Subdomain of EGFR Signaling: Implications for the Anti-Pruritic Activity of NK1R Antagonists

**DOI:** 10.3390/medicines6040114

**Published:** 2019-12-09

**Authors:** Shawn G. Kwatra, Emily Boozalis, Amy H. Huang, Cory Nanni, Raveena Khanna, Kyle A. Williams, Yevgeniy R. Semenov, Callie M. Roberts, Robert F. Burns, Madison Krischak, Madan M. Kwatra

**Affiliations:** 1 Department of Dermatology, Johns Hopkins University, Baltimore, MD 21287, USA; skwatra1@jhmi.edu (S.G.K.); eboozalis@gmail.com (E.B.); ahuang32@jhmi.edu (A.H.H.); rkhanna8@jhmi.edu (R.K.); kwill184@health.fau.edu (K.A.W.); 2 Department of Anesthesiology, Duke University, Durham, NC 27710, USA; cory.nanni@duke.edu (C.N.); callie.roberts@duke.edu (C.M.R.); robert.burns@duke.edu (R.F.B.); madison.krischak@duke.edu (M.K.); 3 Division of Dermatology, Washington University School of Medicine, St. Louis, MI 63110, USA; yevgeniy.semenov1@gmail.com; 4 Department of Pharmacology and Cancer Biology, Duke University Medical Center, Durham, NC 27710, USA

**Keywords:** aprepitant, erlotinib, pruritus, EGFR, epidermal growth factor receptor, NK1R, neurokinin1-receptor

## Abstract

**Background**: Epidermal growth factor receptor (EGFR) inhibitors can cause serious cutaneous toxicities, including pruritus and papulopustular acneiform skin eruptions. Increasingly, the neurokinin-1 receptor (NK1R) antagonist aprepitant is being utilized as an anti-pruritic agent in the treatment of EGFR-inhibitor induced pruritus. Aprepitant is believed to reduce itching by blocking NK1R on the surface of dermal mast cells. However, the effects of aprepitant on human keratinocytes remains unexplored. **Methods**: Herein, we examine the effects of aprepitant on EGFR stimulation in HaCaT cells using a phosphoproteomic approach including reverse phase protein arrays and Ingenuity Pathway Analysis. Changes in EGFR phosphorylation were visualized using Western blotting and the effect of EGF and aprepitant on the growth of HaCaT cells was determined using the WST-1 Cell Proliferation Assay System. **Results**: We found that aprepitant increased the phosphorylation of EGFR, as well as 10 of the 23 intracellular proteins phosphorylated by EGF. Analysis of phosphoproteomic data using Ingenuity Pathway Analysis software revealed that 5 of the top 10 pathways activated by EGF and aprepitant are shared. **Conclusions**: We propose that aprepitant produces its antipruritic effects by partially activating EGFR. Activation of EGFR by aprepitant was also seen in primary human keratinocytes. In addition to itch reduction through partial activation of shared EGFR pathways, aprepitant exerts a dose-dependent cytotoxicity to epithelial cells, which may contribute to its antitumor effects.

## 1. Introduction

Epidermal growth factor receptor (EGFR) inhibitors such as erlotinib can cause serious cutaneous toxicities, including papulopustular acneiform skin eruptions and severe pruritus [1,2,3]. The skin toxicity of EGFR inhibitors is due to the blockade of EGFR in the epidermis, which was demonstrated by genetic ablation of epidermal EGFR in a mouse model [4]. Similarly, a loss-of-function mutation of EGFR in a human child exhibited skin toxicity resembling that seen in patients taking EGFR inhibitors [5]. 

The effects of erlotinib-induced pruritus on quality of life is substantial; 12–16% of all cancer patients treated with erlotinib develop pruritus, usually within the first few days to weeks of therapy [6]. In addition to its significant effect on psychosocial well-being, pruritus can also interfere with treatment efficacy by leading to poor drug compliance and even dose modifications or discontinuation by healthcare providers [1,7]. A survey of oncologists from 2010 revealed that 76% of practitioners modified a patient’s dose of EGFR inhibitors in response to the associated skin toxicities, and 32% discontinued EGFR inhibitor therapy altogether [8]. Thus, understanding and preventing EGFR inhibitor skin toxicity is critical to improving patient quality of life and survival. 

In recent years, neurokinin-1 receptor (NK1R) antagonists such as aprepitant have emerged as a promising class of medications for the treatment of chronic pruritus [9]. In 2009, a case series in the New England Journal of Medicine first described the successful off-label use of aprepitant to treat severe, recalcitrant itch in three patients with cutaneous T-cell lymphoma (CTCL) [10]. Since then, a case series of cancer patients showed prompt relief of erlotinib-induced itch after administration of aprepitant [11]. Furthermore, a clinical trial in 2012 established the efficacy of aprepitant in reducing pruritus caused by anti-EGFR therapy [12]. The current proposed mechanism for aprepitant’s antipruritic effect is the prevention of the neuropeptide substance P (SP) from binding to NK1R on the surface of dermal mast cells, thus preventing mast cell activation and degranulation [13]. However, this theory remains unconfirmed, and the effect of aprepitant on human keratinocytes remains largely unexplored. 

To better understand the effect of aprepitant on human keratinocytes, we examined the effects of aprepitant on EGFR signaling in HaCaT cells—an immortalized line of human keratinocytes [14]—using reverse phase protein array (RPPA) technology.

## 2. Materials and Methods

Human HaCaT keratinocytes cells were obtained from Dr. Xiao-Fan Wang, Duke University, and were cultured in Dulbecco’s modified Eagle’s medium (DMEM) (Gibco Cat #11960-044), supplemented with 10% fetal bovine serum (FBS) (Sigma, cat #F2442) and 1% L-glutamine (Ionza, cat #17-605E). The HaCaT cell line was authenticated by short tandem repeat (STR) DNA profiling using the Promega GenePrint 10 kit (Promega Cat #B9510) by the Duke University DNA Analysis Facility. The observed STR profile was Amelogenin: (X,Y); CSF1PO: (11,11); D13S317: (11,12); D16S539: (9,9); D5S818: (12,12); D7S820: (9,11); TH01: (9.3,9.3); TPOX: (8,12); vWA: (15,15); D21S11: (28,29). Normal Human Epidermal Keratinocytes (NHEK), isolated from the skin of a 23-year-old female, were purchased from PromoCell GmbH, Heidelberg, Germany (cat #C-12003; lot #401Z028.1). These cells were grown in the media provided by the manufacturer. Primary antibodies to detect total EGFR (cat #2232), EGFR-pY1068 (cat #2234), goat anti-rabbit (cat #7074), and anti-biotin (cat #7075) were purchased from Cell Signaling Technology. Aprepitant was purchased from APExBIO, Houston, TX (cat #A1684). Substance P and epidermal growth factor (EGF) were obtained from Sigma (St. Louis, MO). Human insulin-like growth factor-1 (IGF-1) was purchased from Gibco/ThermoFisher (cat #PHG0078). Erlotinib hydrochloride (cat #E-4007) was purchased from LC laboratories (Woburn, MA, USA). 

### 2.1. Preparation of HaCaT Cells for RPPA Analysis

HaCaT cells (5 × 10^5^) were placed in each well of a six-well plate. The next day, the medium was changed to serum-free. After 24 h in serum-free media, the cells were treated with various drugs. To examine the stimulation of EGFR by EGF, the cells were treated with EGF (100 ng/mL) for 10 min. To examine the blockade of EGF stimulation of EGFR by erlotinib, the cells were treated with erlotinib for 1 h followed by 10 min of exposure to EGF. To study the stimulation of EGFR with aprepitant and other NK1R blockers, cells were treated with these drugs for 1 h. After the treatment, the media was removed and the cells were washed twice with ice-cold PBS. The washed cells were taken in 120 µL of RPPA lysis buffer (1% Triton X-100, 50 mM 4-(2-hydroxyethyl)-1-piperazineethanesulfonic acid (HEPES) pH 7.4, 150 mM NaCl, 1.5 mM MgCl_2_, 1 mM egtazic acid (EGTA), 100 mM NaF, 10 mM Na pyrophosphate, 1 mM Na_3_VO_4_, 10% glycerol, plus a cocktail of protease and phosphatase inhibitors (recipe provided by M.D. Anderson’s Reverse Phase Protein Array core facility)), centrifuged, and processed according to the instructions provided by the RPPA core facility [15].

### 2.2. Ingenuity Pathway Analysis of RPPA Data

Ingenuity pathway analysis (IPA) suite (Qiagen, Germany) was separately run on RPPA data from cells treated with EGF and aprepitant. The top ten canonical pathways affected (ranked by p value from Fisher’s exact test) by these treatments were determined. The “threshold” (vertical dotted line) shows a *p* value of 0.05. The “ratio” (line with points on each bar) refers to the proportion of molecules in the dataset that mapped to IPA’s canonical pathway.

### 2.3. Western Blotting

**Figure 1:**
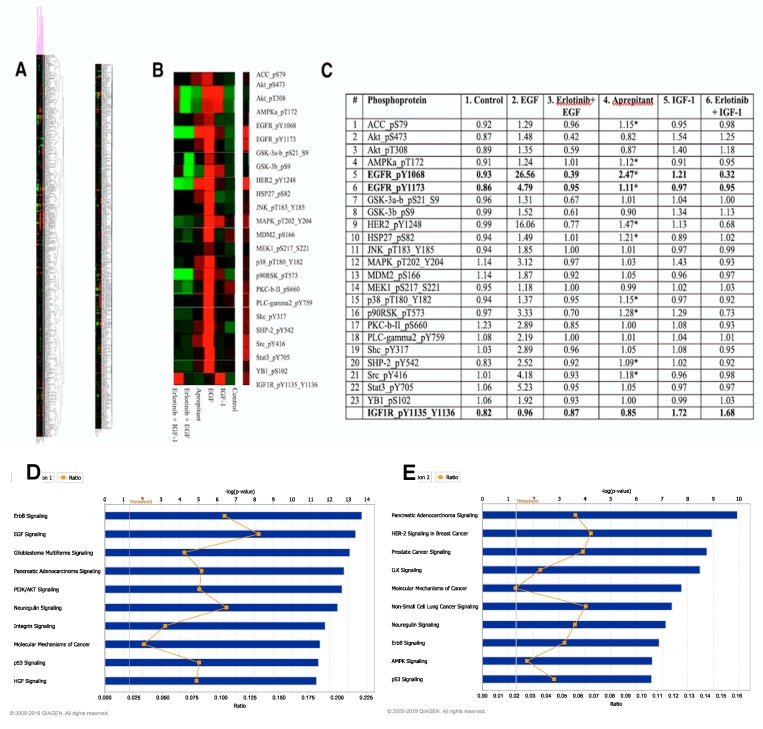
Proteomic analysis of HaCaT cells using reverse phase protein array (RPPA) technology. (**A**) Unsupervised and supervised heatmaps from RPPA analysis on HaCaT cells treated with the following agents: Control (DMSO only), EGF (100 ng/mL) for 10 min, IGF-1 (100 ng/mL) for 10 min, erlotinib (10 µM) for 60 min followed by EGF (100 ng/mL) for 10 min, erlotinib (10 µM) for 60 min followed by IGF-1 (100 ng/mL) for 10 min, aprepitant (10 µM) for 60 min. (**B**) A section of heatmap focusing on intracellular proteins phosphorylated by epidermal growth factor receptor (EGFR) activation. (**C**) List of 23 phosphoproteins whose phosphorylation increased by more than 20% upon stimulation of EGFR by EGF. Phosphorylation of 10 of these proteins (43% of the total phosphorylated upon EGF stimulation) also increased following treatment with aprepitant (marked with an asterisk). (**D**) Top 10 pathways determined by Ingenuity Pathway Analysis of RPPA data from control and EGF-stimulated HaCaT cells. (**E**). Top 10 pathways determined by Ingenuity Pathway Analysis of RPPA data from control and aprepitant-treated HaCaT cells.

Changes in EGFR phosphorylation in HaCaT cells and NHEK primary keratinocytes were visualized using Western blotting (Figure 1A–D) as described previously [15]. Briefly, approximately 500,000 freshly dissociated HaCaT or primary keratinocytes were plated in six-well plates containing 5 mL of media. After 24 h, the media was changed to 5 mL of serum-free media and cells were incubated for one hour with dimethylsulfoxide (DMSO) (control and EGF groups) or with different concentrations of aprepitant in DMSO in a 37 °C, 5% CO_2_ incubator. After this incubation, the cells in one well (EGF group) were treated with 5 μL of 100 μg/mL EGF for 10 min. The media was removed from all wells and cells were washed twice with ice-cold PBS. The washed cell pellets were added to 100 μL of RPPA lysis buffer and the protein concentration was measured, as detailed previously [15]. About 10 µg of lysate proteins from each treatment group was run on a 4–12% NovexBis-Tris gel (Life Technologies, Grand Island, NY, USA). The separated proteins were transferred to a polyvinylidene difluoride membrane, blocked with 5% milk, then probed with a rabbit polyclonal p-EGFR Y1068 antibody (catalog #2234; Cell Signaling Technology, Beverly, MA, USA) or a rabbit polyclonal EGFR antibody to detect total EGFR. Rabbit Beta-Actin antibody was used to show equal protein loading. The blot was developed using the Pierce Enhanced Chemiluminescence (ECL) Western Blotting Substrate Kit (cat #32106, ThermoFisher Scientific, Waltham, MA, USA) and Biomax MR film (Sigma-Aldrich Corp., St. Louis, MO, USA).

### 2.4. Effect of EGF and Aprepitant on the Growth of HaCaT Cells

The effect of EGF and aprepitant on the growth of HaCaT cells was determined using the WST-1 Cell Proliferation Assay System according to the manufacturer’s instructions (cat #MK400Takara Bio USA; Mountainview, CA). Briefly, freshly dissociated HaCaT cells were seeded in a 96-well plate at a density of 2000–5000 cells/well in 200 μL of media. The plates were placed in a cell culture incubator (37 °C, 5% CO_2_) overnight and the media was changed to serum-free media. After 24 h, cells were treated with different concentrations of EGF (dissolved in PBS) and aprepitant (dissolved in DMSO). Each concentration of EGF and aprepitant was tested in quadruplicate. After incubating the cells for 3–4 days in the incubator, 20 μL of WST-1 reagent was added to each well. The cells were again incubated in the incubator for 1–4 h, and absorbance was measured at a wavelength of 450 nm using Biorad’s Bench Mark Plus plate reader (Hercules, CA, USA). Background absorbance was measured by adding the WST-1 reagent to wells containing the media but no cells. The experiment was repeated four times and the data were analyzed using GraphPad Prism 5.0 software (San Diego, CA, USA).

## 3. Results

Figure 1A show shows the results of RPPA analysis on HaCaT cell lysates treated with the following conditions: Control, EGF, IGF-1, erlotinib followed by EGF, erlotinib followed by IGF-1, and aprepitant. Figure 1B shows the RPPA analysis results with a focus on the intracellular proteins that were phosphorylated by EGFR activation. Figure 1C lists the HaCaT human keratinocyte proteins whose phosphorylation increased by at least 20% upon stimulation by EGF. The EGF-induced increase in the phosphorylation of these proteins (Figure 1C, column 2) was mediated through EGFR, because no increase in phosphorylation was seen with EGF when the cells were pre-treated with the EGFR-tyrosine kinase inhibitor (TKI) erlotinib (Figure 1C, column 3). Figure 1C, column 4 shows HaCaT cell proteins whose phosphorylation was increased when exposed to aprepitant. Proteins marked with an asterisk demonstrated an increase in phosphorylation. As can be seen, 10 out of 23 proteins phosphorylated by EGF stimulation were also phosphorylated by aprepitant, albeit not as robustly as EGF. These data indicate that aprepitant in HaCaT cells serves as a partial agonist of EGFR. Interestingly, cross-talk between EGFR and NK1R was also reported in human mesenteric preadipocytes, but in these cells EGFR phosphorylation was increased by substance P (SP), an agonist of NK1R [16]. There are additional examples of SP increasing the phosphorylation of EGFR [17,18]. However, none of these reports came from keratinocytes. To our knowledge, the keratinocyte is the only cell type where EGFR phosphorylation is increased by an NK1R antagonist. The mechanism by which NK1R blockade, rather than stimulation, in keratinocytes increases EGFR phosphorylation remains to be determined. However, our preliminary data indicate that keratinocytes express only the truncated isoform of NK1R (Kwatra et al., unpublished data). 

We also treated HaCaT cells with IGF-1, because IGF-1 was implicated in the transmodulation of EGFR in keratinocytes [19]. As expected, IGF-1 stimulation of HaCaT cells increased the phosphorylation of IGF-1R at Y1135 and Y1137 (last row in Figure 1C) indicating that IGF-1R in HaCaT cells was functional. IGF-1 also increased the phosphorylation of EGFR (visualized in columns 1 and 5). Further, IGF-1 increased the phosphorylation of p90RSK_T543, which was blocked by erlotinib. Thus, our data showed that a downstream kinase of EGFR signaling was activated by IGF-1 and was blocked by erlotinib. Taken together, our results provide direct evidence of IGF-1 activation of EGFR in keratinocytes, which was suggested by previous reports [16]. However, the increase in EGFR signaling by IGF-1 was much less than that seen with aprepitant (compare columns 4 and 5).

To obtain further insight into aprepitant’s mechanism of action, Ingenuity Pathway Analysis software was used to compare the top ten pathways activated by EGF (Figure 1D) and aprepitant (Figure 1E). These data show that five of the top ten signaling pathways activated by EGF and aprepitant are shared: ErbB, Pancreatic Adenocarcinoma, Neuregulin, Molecular Mechanisms of Cancer, and p53. 

**Figure 2:**
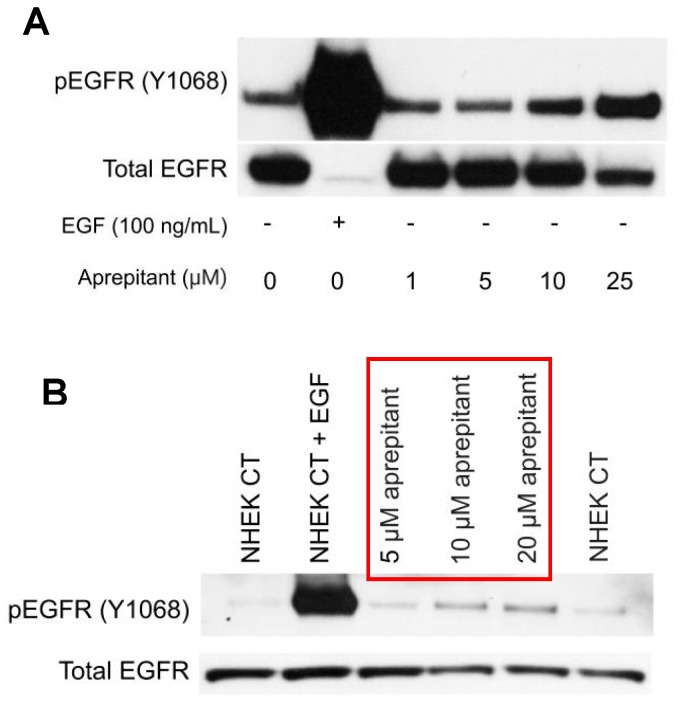
Visualization of EGFR phosphorylation at Y1068 by Western blotting. (**A**) HaCaT cells were treated with different concentrations of aprepitant. Western blot analysis showed that aprepitant stimulated the phosphorylation of EGFR in a dose-dependent manner. (**B**) Normal Human Epidermal Keratinocytes (NHEK) cells were treated with different concentrations of aprepitant. Western blot analysis showed that aprepitant increased the phosphorylation of EGFR in primary keratinocytes in a dose-dependent manner, similar to that seen in HaCaT cells.

To confirm the observed aprepitant-induced increase in EGFR phosphorylation seen with RPPA analysis (Figure 1C), Western blotting was utilized (Figure 2). As Figure 2A shows, aprepitant increased the phosphorylation of EGFR in a dose-dependent manner. Note that the antibody that was used for total EGFR (catalog #2232, Cell Signaling Technology, Danvers, MA, USA) was raised against a peptide from an EGFR sequence that included Y1068; therefore, it did not recognize EGFR when it was phosphorylated at Y1068 (this explains why we had a weaker band for total EGFR when the receptor was phosphorylated). 

We next examined whether aprepitant-induced EGFR activation seen in HaCaT cells, a cell line derived from human keratinocytes, was also seen in primary human keratinocytes (NHEK) cells. As Figure 2B shows, aprepitant also stimulated the phosphorylation of EGFR in NHEK cells in a dose-dependent manner. 

**Figure 3:**
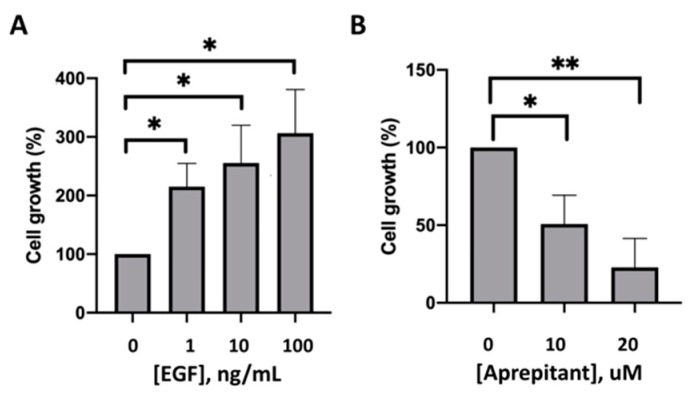
Effects of EGF and aprepitant on growth of HaCaT Cells. (**A**) HaCaT cells treated with EGF showed a significant dose-dependent increase in cell proliferation compared to incubation with PBS alone. (**B**) HaCaT cells treated with aprepitant (AP) showed a significant dose-dependent significant increase in cell death compared to incubation with DMSO alone. (* indicates *p* < 0.05, ** indicates *p* < 0.01).

Finally, the effects of aprepitant on cell division, as measured by the WST-1 Cell Proliferation Assay, were tested by incubating HaCaT cells with different concentrations of aprepitant and EGF, respectively. As expected, HaCaT cells demonstrated a significant dose-dependent increase in cell proliferation upon incubation with EGF as compared to PBS (Figure 3A). In contrast, HaCaT cells showed a significant dose-dependent cell death with increasing concentrations of aprepitant (Figure 3B). 

## 4. Discussion

A key finding of our study was that aprepitant activated EGFR in human keratinocytes, a novel finding that may explain aprepitant’s anti-pruritic activity. Despite partial activation of EGFR in keratinocytes, aprepitant also demonstrated dose-dependent cytotoxicity to epithelial cells in our study that was consistent with previous reports of its anti-tumor effects [20,21,22]. One hypothesis for this phenomenon is that the truncated form of NK1R may predominate in human skin, in addition to being overexpressed in tumor cells. In contrast, the full-length form of NK1R is typically expressed in normal non-tumor cells. This difference may explain the dose-dependent toxicity of aprepitant that was observed in HaCaT cells. It should be noted, however, that aprepitant-induced cytotoxicity should be negligible at doses lower than 10 µM that are used for anti-pruritic effects. 

The cutaneous reactions seen in erlotinib-treated patients appear to be clinical indicators of treatment response, with the severity of cutaneous toxicities also appearing to be dose-dependent [6,23]. There is also a strong positive correlation between the severity of cutaneous toxicity following EGFR inhibition and overall patient survival [24]. Thus, despite the adverse effects on quality of life and compliance, the presence of cutaneous symptoms in these cancer patients may be viewed as a positive event. Therefore, aprepitant may be recommended as a therapeutic option for management of EGFR-TKI-induced itch [4]. 

The pathophysiology of tyrosine-kinase inhibitor-induced pruritus is incompletely understood, and data studying this phenomenon are sparse [23]. It is important to understand the mechanism underlying EGFR inhibitor-induced pruritus and skin toxicity to prevent premature termination of chemotherapy and to improve quality of life in cancer patients. Future studies should be directed at further understanding the mechanism of EGFR-TKI-induced pruritus and skin toxicity in order to better develop pharmacotherapies to relieve symptoms without interfering with cancer treatment. 

In summary, our findings demonstrated that aprepitant activated EGFR in human keratinocytes by interacting with NK1R, and this might be the mechanism by which aprepitant reduces erlotinib-induced pruritus and skin toxicity. 

We also showed that, in addition to partial activation of EGFR that may mediate its antipruritic effects, aprepitant also displayed antitumoral effects in suppressing cell growth. Future research on EGFR signaling and skin cytotoxicity in patients receiving the U.S. Food and Drug Administration (FDA)-approved doses of aprepitant is needed to verify the effects of aprepitant on human keratinocytes in vivo.
